# Sulfobetaine Cryogels for Preferential Adsorption of Methyl Orange from Mixed Dye Solutions

**DOI:** 10.3390/polym13020208

**Published:** 2021-01-08

**Authors:** Ramona B. J. Ihlenburg, Anne-Catherine Lehnen, Joachim Koetz, Andreas Taubert

**Affiliations:** Institute of Chemistry, University of Potsdam, Karl-Liebknecht-Str. 24-25, Building 26, D-14476 Potsdam, Germany; rihlenbu@uni-potsdam.de (R.B.J.I.); lehnen@uni-potsdam.de (A.-C.L.); koetz@uni-potsdam.de (J.K.)

**Keywords:** cryogel, water treatment, dye removal, methyl orange, methylene blue, dye mixture

## Abstract

New cryogels for selective dye removal from aqueous solution were prepared by free radical polymerization from the highly water-soluble crosslinker N,N,N’,N’-tetramethyl-N,N’-bis(2-ethylmethacrylate)-propyl-1,3-diammonium dibromide and the sulfobetaine monomer 2-(N-3-sulfopropyl-N,N-dimethyl ammonium)ethyl methacrylate. The resulting white and opaque cryogels have micrometer sized pores with a smaller substructure. They adsorb methyl orange (MO) but not methylene blue (MB) from aqueous solution. Mixtures of MO and MB can be separated through selective adsorption of the MO to the cryogels while the MB remains in solution. The resulting cryogels are thus candidates for the removal of hazardous organic substances, as exemplified by MO and MB, from water. Clearly, it is possible that the cryogels are also potentially interesting for removal of other compounds such as pharmaceuticals or pesticides, but this must be investigated further.

## 1. Introduction

Cryogels have been known since the 1970s and are generally made by polymerization around ice crystals [1,2]. As the ice crystals are rather large, the resulting cryogels contain interconnected pores in the micrometer range. The large pores result in a good accessibility for fluids or gases and cryogels have therefore been used for cell storage, tissue engineering, bone regeneration, and drug delivery [2,3].

Besides application in the biomedical and biomaterials fields, water treatment with cryogels has also attracted attention [1,4,5,6,7]. For example, Evli et al. [8] used acetylcysteine modified cryogels to remove Zn(II), Cd(II), and Pb(II) from water. All ions were removed with an efficiency of over 90%. Bilgin et al. [9] studied the removal of complex mixtures of heavy metals from a series of industrial wastewater sources using nicotinamide decorated poly(2-hydroxyethyl methacrylate-glycidyl methacrylate) cryogels. According to the authors, the removal of different metal cations depends on whether or not the parent polymer matrix is modified with nicotinamide or not. In some cases, the removal efficiency is higher with the nicotinamide modification, notably Zn(II), Al(III), or Cu(II), while in some cases such as Hg(II) or Pb(II), the unmodified cryogels are more effective. Using a related approach, Huseynli et al. [10] employed metal cation imprinted cryogels to remove Cd(II) from water. Again, the authors used methacrylate polymers modified with cysteine methyl ester residues to generate cryogels with Cd(II) removal efficiencies reaching more than 98%. Sarkaya et al. [11] used an analogous approach to imprint poly(hydroxyethyl methacrylate) cryogels modified with N-methacryloyl-L-cysteine with Ag^+^ and subsequently removed over 70% of Ag^+^ ions present in test solutions. In contrast, the non-modified cryogels (i.e., cryogels without pendant L-cysteine groups) only removed below 1% of the Ag^+^ present. Overall, these and many other studies show that cryogels (imprinted or not) can be effective media for heavy metal removal [1,6].

Organic dyes have attracted tremendous attention as well in water treatment. This is mainly due to the fact that many dyes are endocrine disruptors [12]. This is a severe problem, and the US National Institute of Health has precisely summarized the issues with endocrine disruptors: “Many chemicals, both natural and man-made, may mimic or interfere with the body’s hormones, known as the endocrine system. Called endocrine disruptors, these chemicals are linked with developmental, reproductive, brain, immune, and other problems. Endocrine disruptors are found in many everyday products, including some plastic bottles and containers, liners of metal food cans, detergents, flame retardants, food, toys, cosmetics, and pesticides. Some endocrine-disrupting chemicals are slow to break-down in the environment. That characteristic makes them potentially hazardous over time [13].”

MO and MB, along with many other organic dyes, therefore pose a severe threat to plant, animal, and human health [14]. Among others, this is due to the fact that they are highly water soluble and can thus be transported in virtually all environments. According to El-Geudi, 2% of all manufactured dyes are released into water bodies at manufacturing sites [15]. As a result of the rather large dye concentrations and their adverse health effects such as neurological damage [16], there is a need for cheap, rapid, and effective methods for dye removal from all kinds of water sources. This particularly applies to developing countries [17].

Indeed, cryogels have already been used for dye removal. For example, Uyar et al. [18] have shown that methylene blue (MB) can be removed effectively from water via a composite alginate/clay cryogel. Similarly Ul’yabaeva et al. [19] demonstrated the effective removal of acid yellow K with a chitosan/poly(vinyl alcohol) cryogel. Chen et al. [20] demonstrated that the combination of a cryogel with a photocatalyst is an effective tool for the removal of organic dyes. Specifically, these authors produced nanocellulose cryogels with a positively charged, quaternized surface and N-doped titania for the removal and degradation of methyl orange (MO). Similarly, Sahiner et al. [21] demonstrated that graphene oxide/polymer composite cryogels are effective for MO removal as well.

The current study focuses on a new, sulfobetaine-based cryogel for dye removal from aqueous solution. In particular, the study presents the first evidence of charge-dependent separation of organic dyes using a betaine cryogel. Using a 1:1 mixture of MB and MO as a model system, the cryogels can be used for selective MO removal from aqueous solution. This is thus the first demonstration of selective dye removal from water with a cryogel.

## 2. Results and Discussion

**Figure 1:**
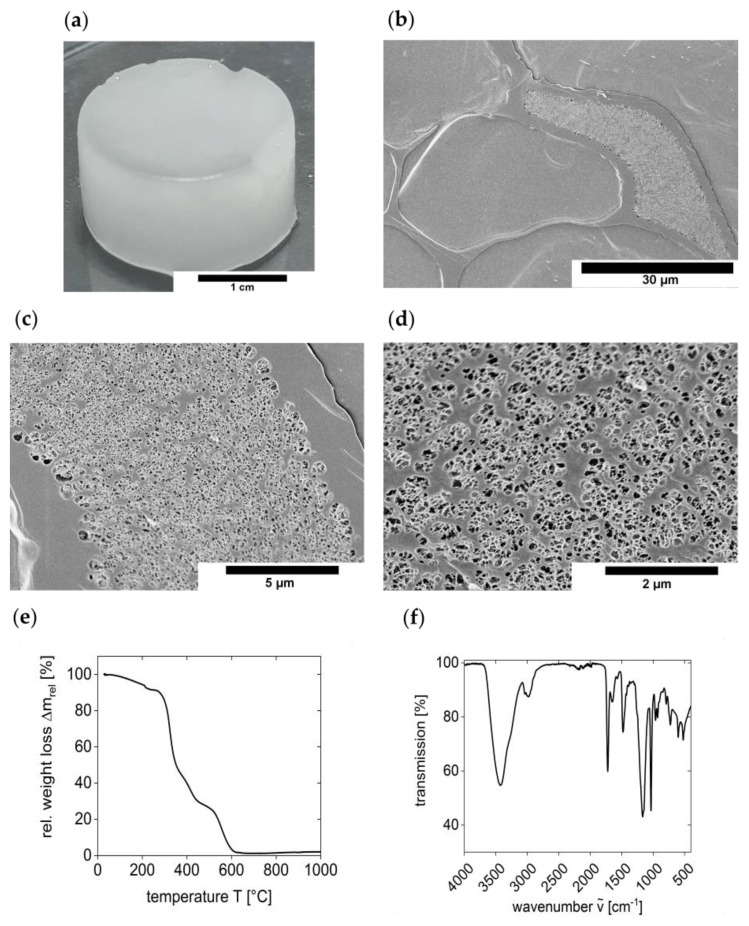
(**a**) Photograph of a cryogel, (**b**–**d**) SEM-images of the cryogel at different magnifications, (**e**) TGA data, and (**f**) IR spectrum of the cryogel.

The cryogels were synthesized from the highly water-soluble crosslinker N,N,N’,N’-tetramethyl-N,N’-bis(2-ethylmethacrylate)-propyl-1,3-diammonium dibromide [22] and the sulfobetaine monomer 2-(N-3-sulfopropyl-N,N-dimethyl ammonium)ethyl methacrylate using a redox-initiated free radical polymerization with potassium persulfate (KPS) and tetramethylethylenediamine (TMEDA) in water. Upon polymerization at −32 °C for three days, a white, opaque, and stable solid is obtained, Figure 1a. The shape can be molded by choice of the reaction vessel and the samples typically used in this study have a weight of 4.63 ± 0.15 g, a diameter of 2.3 ± 0.1 cm, and a height of 1.3 ± 0.1 cm after swelling.

Cryo-scanning electron microscopy (cryo-SEM) of the material (Figure 1b,d) shows a network with large pores in the micrometer range. The pores have irregular but typically elongated shapes of more than 30 µm in length and about 15 µm in width. Moreover, some of these larger features also exhibit smaller, roughly spherical features with diameters around 1 µm. These smaller pores are reminiscent of the structure of hydrogels that were synthesized via free radical polymerization at 70 °C (rather than via cryogel synthesis) [22].

The water content of the swollen cryogels is about 87% as determined via thermogravimetric analysis (TGA). TGA (Figure 1e) of dry cryogels shows an additional mass loss of ca. 2% between room temperature and 120 °C. This additional weight loss is, however, hard to assign to loss of water alone because the TGA data do not show a clear step in this temperature range. As a result, the mass loss up to ca. 200 °C (where a more significant weight loss begins) may stem from the evaporation of residual water and the beginning cryogel decomposition.

Figure 1f shows a representative infrared spectrum of a dried cryogel. O–H stretching vibration of remaining water is visible as a broad band at 3429 cm^−1^ and a medium sharp band around 1650 cm^−1^. Bands at 3039 and 2981 cm^−1^ can be assigned to an asymmetric N-H stretching vibration or a C–H stretching vibration of unreacted double bond in monomers. An additional band at 1167 cm^−1^ correspond to the C–N stretching vibration. A sharp and strong band at 1721 cm^−1^ indicates the presence of carbonyl groups and a band at 1034 cm^−1^ is indicative of the presence of SO_3_^−^ groups. All other bands at 1479 cm^−1^ and in the range of 963–522 cm^−1^ stem from C–H and C–C bond vibrations of the polymer network.

**Figure 2:**
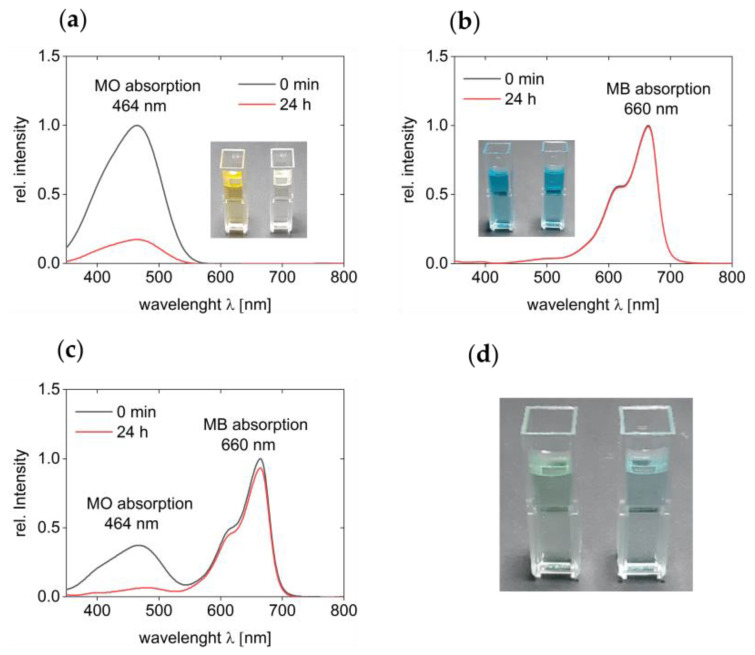
UV–VIS spectra of dye solutions. (**a**) Spectra of MO solutions before the adsorption experiment (0 min; black line) and after 24 h (red line) of incubation with cryogel, (**b**) spectra of MB solutions before and after the experiment. Individual spectra of both measurements as individual spectra are shown in Appendix B, (**c**) spectra of 1:1 solutions containing both MO and MB before and after adsorption. All insets show cuvettes before (cuvette on the left) and after adsorption (cuvette on the right). (**d**) Photograph of cuvettes containing the MO/MB mixtures before (left) and after (right) the adsorption experiment.

Quantification of dye adsorption by the cryogel was done with UV–VIS spectroscopy, Figure 2. Initially, two separate solutions of MO and MB with a concentration of 50 mg/L each were used to evaluate the sorption capability of the cryogel. Figure 2a shows that MO has an absorption maximum at 464 nm in aqueous solution. The intensity of this band is strongly reduced after 24 h of exposure of the solution to the cryogel, and a quantitative analysis shows that 97% of MO are removed from the solution within 24 h. This is also visible by the discoloration of the solutions in the cuvettes and reaction flasks.

In contrast, the cryogel does not adsorb MB in significant amounts, Figure 2b. The spectra of these solutions show an absorption maximum at 660 nm and this band does not change over the course of 24 h. This indicates that essentially no MB is removed. Again, visual inspection confirms this as the intense blue color of the aqueous solution remains the same before and after the adsorption experiment.

From UV–VIS spectroscopy and proper calibration, the actual mass of material removed from the solution can be calculated (see experimental part for details). In the case of MO, the mass of MO in solution is reduced from an initial 2.0 mg at the beginning of the experiment to 0.3 mg at the end of the experiment. This corresponds to 85% of the dye removed from solution. In the case of MB, only 0.2 mg (8%) disappear within 24 h. Overall, these data clearly show a significant difference between MO and MB uptake by the cryogels.

**Figure A1:**
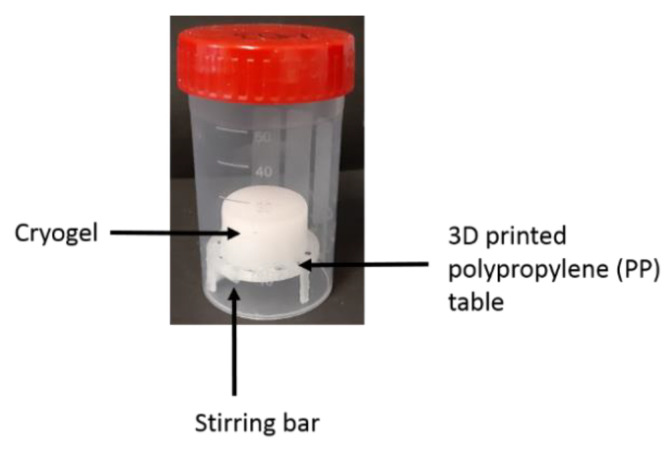
Photograph of the experimental set-up during adsorption measurement. It contains a cryogel, 3D-printed polypropylene (PP) table, and a stirring bar. 40 mL of organic dye solution were added for the measurement.

It must be noted, however, that also the cryogel support (see Appendix A) does adsorb some dye. The cryogel support was used to avoid damage of the cryogel by the stir bar. It was made by 3D printing (fused deposition modeling) of poly(propylene) (PP) using conditions as noted in the experimental section. Adsorption experiments using the same approach as just described but using only the cryogel support (Figure A1, Appendix A, no cryogel present in the system) show that 4% of MO and 5% of MB are adsorbed by the PP support structure and the reaction vessel. As a result, the true adsorption capacity of the cryogel must be corrected for the adsorption by the cryogel support and the vial. Consequently, the corrected values are (85-4) = 81% of MO and (8-5) = 3% of MB that are taken up by the cryogel under identical conditions.

Finally, Figure 2c shows the same data for a mixture of MO and MB. Clearly, UV–VIS spectra of solutions taken after 24 h of incubation with the cryogel show that only the band at 464 nm (MO) is drastically reduced in intensity. In contrast, the intensity of the band a 660 nm (MB) only shows a minute loss in intensity, consistent with the above data. The solutions before and after treatment with the cryogel show a turquoise tint before and an intense blue color after treatment, Figure 2d, indicating that MO is indeed removed from the solution, while MB is not.

**Figure 3:**
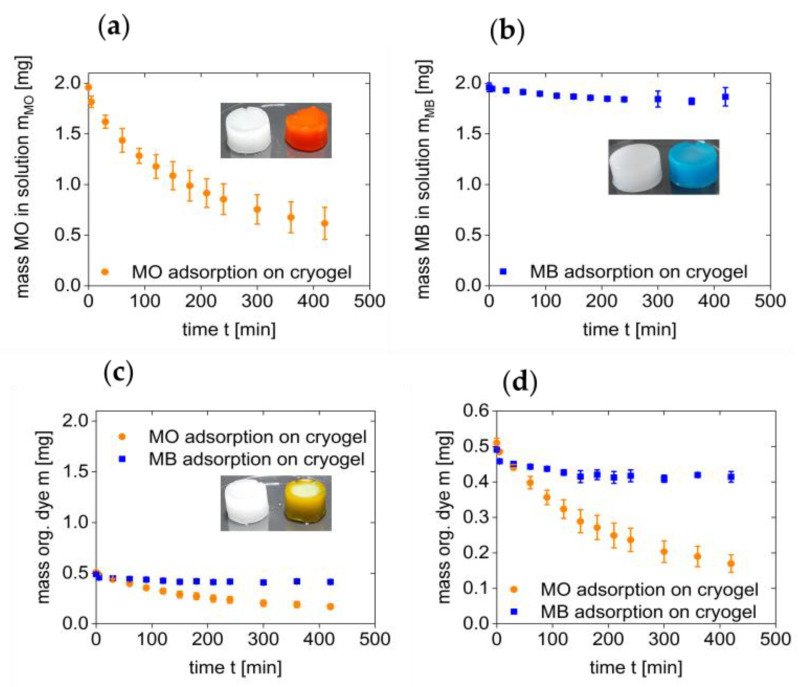
Mass of organic dye in solution vs. treatment time. (**a**) MO, (**b**) MB, (**c**) MO/MB mixture. Panel (**d**) is an enlarged section of the data shown in panel (**c**) to demonstrate the details of the two curves. Insets show cryogels before (left) and after (right) the experiment (24 h of exposure). Note that for the measurements of the dye mixtures (data in panels (**c**,**d**)) the concentrations of both dyes had to be reduced to 12.5 mg/L each. At higher dye concentration in the mixtures, the dyes precipitate and produce a turbid liquid. In these systems reproducible measurements are not possible.

Figure 3a shows the mass loss (mass removed from solution) vs. exposure time for the first 7 h of the experiment. Consistent with the above data, MO removal is relatively fast and the within the first 200 min of the process, the mass of MO in solution is reduced to half the initial amount. This is followed by a slower decrease to ca. 0.75 mg after 7 h. In contrast, and consistent with the data shown above, the mass of MB in solution remains essentially constant and no significant reduction can be observed over time, Figure 3b.

As there is a strong preference of the cryogel for MO, the cryogels were also evaluated for their preferential removal of MO from a MO/MB mixture, Figure 3c,d. The UV–VIS spectra of the mixtures are a combination of the two individual spectra (Figure 2c) and the two components can thus easily be monitored independently. Clearly, the cryogel reduces the mass of the MO in solution by 85% from 0.50 to 0.08 mg. In contrast, the mass of MB is only reduced by 0.05 mg (10%). These data are thus perfectly consistent with the behavior of the individual measurements above and are again qualitatively supported by visual inspection of the color of the solutions and the colors of the cryogels at the end of the experiment. Specifically, the cryogels used for the treatment of the MO/MB mixtures are orange with a slight blue hue, again indicating a highly preferential uptake of MO. Table 1 summarizes the results.

Table 2 shows the corresponding sorption capacities calculated from the above data after 7 and 24 h of exposure. Clearly, the cryogels show a higher sorption capacity for MO than for MB. This is further supported by the optical appearance; see Figure 3c.

Clearly, it would be interesting to compare the results with findings from other studies. However, as stated in the introduction, the number of studies on the subject is rather limited and some of the studies used different dyes [19], which further complicates a comparison. Our data can thus only be compared to a few other datasets [18,20,21,23], where MO or MB was removed with different cryogels from aqueous solution. The removal rates in these studies were between 48 and 99% for both MB and MO. This shows that the current materials are comparable with these previously reported materials but have the advantage that they are (1) selective for MO and (2) are much simpler as far as their chemical composition goes. Only the materials reported in Reference [21] show a similar selectivity but with the tradeoff that the material is much more complex. Only very recently a further study has presented MB selective cryogels made from dextran, i.e., the exact inverse of the current materials [24]. 

## 3. Conclusions

In summary, the current study shows that sulfobetaine cryogels are effective adsorbents for MO but much less so for MB. Although the exact mechanism of dye adsorption is not known at the moment, the fact that one of the dyes (MO) is negatively charged while the other dye (MB) is positively charged may be a major factor in these studies. Considering the chemical structure of the cryogel, which is based on a sulfobetaine monomer and a di-cationic crosslinker, it is likely that anionic dyes have a higher chance of being taken up by the cryogel. In spite of this, the high fraction of MO uptake is quite surprising and may possibly also be related to details of the internal structure of the cryogels. As stated in our previous study, [22] one of the main advantages of the sulfobetaine groups is the fact that they tend to stabilize the hydrogel structure via numerous ionic interactions thus providing stable materials that have the potential for application in water remediation.

Finally, while the sorption capacities of the current material can still be improved, the current cryogels show an additional feature, selective dye removal, which has not been reported for cryogels before. As a result, the current materials are prototypes for advanced, selective adsorbent that may find application in water treatment, but possibly also in chromatography or liquid management.

## 4. Materials and Methods

**Materials**. 2-Dimethylaminoethyl)methacrylate (DMAEMA, stabilized with hydrochinone monomethylether for synthesis, Merck, Darmstadt, Germany), 1,3-dibrompropane (98%, Alfa Aesar, Kandel, Germany), dimethyl formamide (DMF, water < 150 ppm, VWR Prolabo, Darmstadt, Germany), acetone (GPR RECTAPUR^®^, VWR, Darmstadt, Germany), tert-butyl methyl ether (99%, Alfa Aesar, Kandel, Germany), 3-[dimethyl-[2-(2-methylprop-2-enoyloxy)ethyl]azaniumyl]propane-1-sulfonate (SPE, Merck, Darmstadt, Germany), potassium peroxydisulfate (≥99%, Fluka Analytical, München, Germany), tetramethylethylenediamine (TMEDA, Reagen Plus^®^ 99%, Sigma Aldrich, Darmstadt, Germany), methylene blue (C.I. 52015, AppliChem, Darmstadt, Germany), methyl orange (C.I. 13025 ACS, Reag. PH Eur., Merck, Darmstadt, Germany), and polypropylene filament (Ultimaker PP) were used without further purification.

**Crosslinker synthesis**. The crosslinker N,N,N’,N’-tetramethyl-N,N’-bis(2-ethylmethacrylat)-propyl-1,3-diammonium dibromide (TMBEMPA/Br) was synthesized as described previously [22]. In short, 2 equivalents of DMAEMA and 1 equivalent of 1,3-dibromopropane were dissolved in 20 mL of DMF. The reaction mixture was stirred at 30 °C for 30 min and then stirred overnight at room temperature. The resulting white solid was washed two times with 250 mL of acetone and two times with 250 mL of MTBE and then dried under high vacuum overnight, yielding a white solid (75% yield). Analysis was consistent with previous analytical results.

**Crosslinker characterization**. Melting point: 76.7 °C (onset of DSC signal). CHN analysis: experiment (calculated) C: 43.64% (44.20%), H: 6.94% (7.03%), N: 6.43% (5.43%). 1H-NMR (300 MHz, D_2_O) δ (ppm): 1.88 (s, 3-H), dqtt, J = 6.22; 5.27; 5.27; 5.27; 3.58; 3.58; 2.64; 2.64 Hz, 9-H); 3.21 (s, 6-H, 7-H, 11-H, 12-H), 3.42–3.56 (m, 8-H, 10-H), 3.81 (dt, J = 4.66; 2.28 Hz, 5-H, 13-H), 4.6 (br. s., 4-H, 14-H), 5.67–5.78 (m, 2-H, 16-H), 6.1 (d, J = 0.94 Hz, 1-H, 17-H). 13C-NMR (300 MHz, D_2_O) δ (ppm): 16.5, 17.9, 52.3, 59.2, 62.9, 64.3, 125.2, 136.0, 167.2. MS (ESI in water) (m/z): [M^+^] calc. for C_19_H_36_Br_2_N_2_O_4_ 516.10, found 435.18 for C_19_H_36_BrN_2_O_4_. Note: only a species with one bromide was observed. ATR-IR (diamond, 298 K, ($\tilde{v}$, cm^−1^)): 3449, 3384, 3237, 3019, 2963, 1718, 1634, 1473, 1453, 1427, 1404, 1370, 1320, 1295, 1172, 1043, 1030, 1012, 955, 918, 897, 865, 815, 661, 567, 471.

**Cryogel synthesis**. For cryogel synthesis, the crosslinker TMBEMPA/Br (0.12 mmol, 0.0612 g) and the monomer SPE (4 mmol, 1.1175 g) were dissolved in 2.6 mL of distilled water. After purging with nitrogen for 30 s, the polymerization catalyst TMEDA (50 µL) was added. The initiator KPS (0.02 mmol, 5.4 mg) was dissolved separately in 1 mL of d.i. water. After combining the two precursor solutions, 3 mL of the reaction mixture were transferred to a bottle with rolled rim and snap-on lid and placed where in a refrigerator for three days at −32 °C.

**Figure A3:**
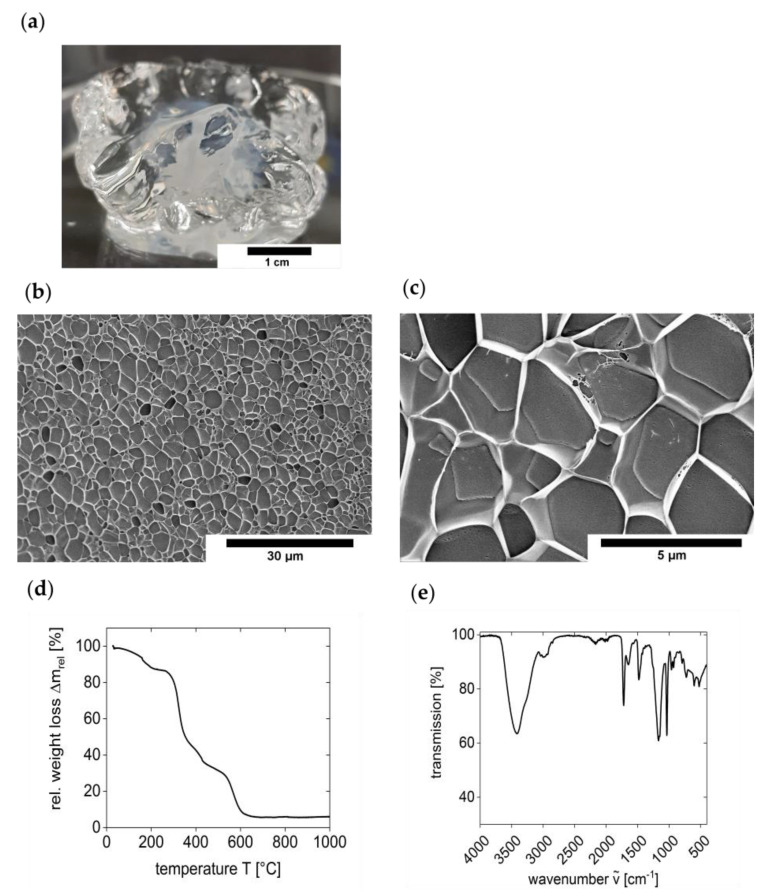
(**a**) Photograph of a hydrogel obtained by polymerization at room temperature after 20 min, (**b**,**c**) cryo-SEM images of the hydrogel at different magnifications, (**d**) TGA data, and (**e**) IR spectrum of the same hydrogel.

*Note*: the same synthesis can also be done at room temperature. Unlike the cryogels, the materials resulting from these reactions are not dimensionally stable; see Figure A3 (Appendix C) for details.

**Water content**. For determination of water content in cryogel, a gravimetric approach were done in first step with a compartment drier (Memmert UF55Plus with grating, opened system setup, ventilation 30%, T = 40 °C, t = 24 h). Quantification of remaining water was done via thermogravimetric analysis (Linseis STA PT-1600, compressed air, 10 K/min, 21–1000 °C).

**Adsorption measurements**. All adsorption experiments were done in poly(propylene) screw cap vials. A 3D printed sample holder (Figure A1, Appendix A) was made from polypropylene (PP) prepared with an Ultimaker 3 (Ultimaker, Utrecht, The Netherlands) via fused deposition modelling with Cura Software 4.5. With a nozzle diameter of 0.4 mm and a printing temperature of 205 °C, a sample with layer height of 0.1 mm with print speed of 25 mm/s was printed on an 85 °C build plate.

This sample holder was used to hold the cryogel away from the stir bar to avoid mechanical damage.

In the adsorption experiments, 40 mL of a 50 mg/L parent solution of MO, MB, or a 1:1 mixture of MO and MB were added. After defined time intervals, 200 µL of the reaction solution were transferred to a poly(methyl methacrylate) (PMMA) cuvette (semi-micro, VWR), and 1.8 mL of ultrapure water were added. For determination of the adsorption capacities of the cryogel, a UV-1900 spectrophotometer (Shimadzu) was used. All spectra were measured from 1000–250 nm with 1 nm sampling interval in the absorbance mode. All measurements were done as a triplex.

Additionally, measurements with only magnetic stirring bar and PP table were done as blank measurement to determine the adsorption of the dyes by the vessel and the PP support table. For calculation of the total amount of organic dye in the solution, a calibration curve with seven points and linear regression with an R^2^ of >0.999 was used.

## Figures and Tables

**Table 1:** Mass of dye in solution at the beginning of the measurement (0 h), after 7 h, and after 24 h.

	Individual Solution		Mixture 1:1	
Time [h]	Mass MO in Solution m_MO_ [mg] ^1^	Mass MB in Solution m_MB_ [mg] ^1^	Mass MO in Solution m_MO_ [mg] ^1^	Mass MB in Solution m_MB_ [mg] ^1^
0	1.96 ± 0.02	1.96 ± 0.04	0.51 ± 0.01	0.49 ± 0.01
7	0.62 ± 0.16	1.87 ± 0.09	0.18 ± 0.03	0.45 ± 0.02
24	0.30 ± 0.09	1.79 ± 0.01	0.08 ± 0.02	0.45 ± 0.01

^1^ raw data without correction for adsorption by vial ad PP table.

**Table 2:** Sorption capacity determined after 7 and after 24 h.

	Individual Solution		Mixture 1:1	
Time [h]	MO Sorption Capacity q [mg/g]	MB Sorption Capacity q [mg/g]	MO Sorption Capacity q [mg/g]	MB Sorption Capacity q [mg/g]
7	0.278 ± 0.039	--- ^1^	0.070 ± 0.006	0.011 ± 0.004
24	0.345 ± 0.024	0.012 ± 0.008	0.090 ± 0.003	0.004 ± 0.002

^1^ Within the experimental error of the measurement, no data could be recorded due to very weak sorption capacities. Note that for the measurements of the dye mixtures (Figure 3c,d), the concentrations of both dyes had to be reduced to 12.5 mg/L each. At higher dye concentration in the mixtures, the dyes precipitate and produce a turbid liquid. In these systems, reproducible measurements are not possible. Moreover, the absorption band at 660 nm (MB) is much more intense, and higher concentrations lead to very high absorption that cannot be quantified anymore.

**Figure A2:**
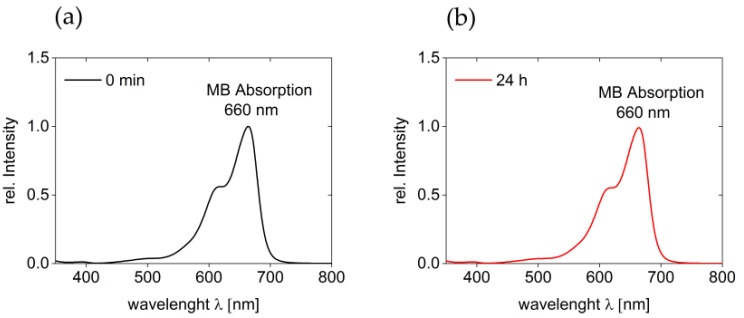
Individual spectra of MB solution at the (**a**) beginning of experiment at 0 min and at the (**b**) ending of the experiment after 24 h.

## Data Availability

The data presented in this study are available on request from the corresponding author.

## Appendix B

Individual spectra of MB solutions before and after the experiment. The spectra are essentially identical and thus overlap completely in Figure 2b.

## Appendix C

**Synthesis of gels at room temperature**. The general gel synthesis is identical to the approach described in the experimental section. The only difference is the temperature and time for polymerization: While the cryogels described in the main body of the article were obtained by polymerization at −32 °C for three days, polymerization at room temperature produces a translucent, colorless, and rather soft hydrogel with no defined outer shape already after 20 min. In contrast to the cryogels, which show regular swelling and a defined shape, these materials show no controlled outer shape even after swelling. The water content of these hydrogels is at least 97%, but their softness and irregular shape makes it rather difficult to perform experiments under controlled conditions. In spite of this, the gels show large and rather uniform pores in cryo-SEM and the TGA and IR data are comparable to the data shown in the main text (Figure 1), Figure A3.
